# Consequences of *Spiraea tomentosa* invasion in Uropodina mite (Acari: Mesostigmata) communities in wet meadows

**DOI:** 10.1007/s10493-024-00951-2

**Published:** 2024-08-01

**Authors:** Blanka Wiatrowska, Przemysław Kurek, Tomasz Rutkowski, Agnieszka Napierała, Paweł Sienkiewicz, Jerzy Błoszyk

**Affiliations:** 1 Department of Botany and Forest Habitats, Poznań University of Life Sciences, Wojska Polskiego 71D, Poznań, 60-625 Poland; 2 Department of Plant Ecology and Environmental Protection, Adam Mickiewicz University, Uniwersytetu Poznańskiego 6, Poznań, 61-614 Poland; 3 Natural History Collections, Adam Mickiewicz University, Uniwersytetu Poznańskiego 6, Poznań, 61-614 Poland; 4 Department of General Zoology, Adam Mickiewicz University, Uniwersytetu Poznańskiego 6, Poznań, 61-614 Poland; 5 Department of Entomology and Environmental Protection, Poznań University of Life Sciences, ul. Dąbrowskiego 159, Poznań, 60-594 Poland

**Keywords:** Uropodina, Alien shrub, Arthropods, Biodiversity, Invasive species, Soil biota

## Abstract

Vegetation cover has been consistently reported to be a factor influencing soil biota. Massive spreading of invasive plants may transform native plant communities, changing the quality of habitats as a result of modification of soil properties, most often having a directional effect on soil microorganisms and soil fauna. One of the most numerous microarthropods in the litter and soil is Acari. It has been shown that invasive plants usually have a negative effect on mites. We hypothesized that invasive *Spiraea tomentosa* affects the structure of the Uropodina community and that the abundance and species richness of Uropodina are lower in stands monodominated by *S. tomentosa* than in wet meadows free of this alien species. The research was carried out in wet meadows, where permanent plots were established in an invaded and uninvaded area of each meadow, soil samples were collected, soil moisture was determined and the mites were extracted. We found that Uropodina mite communities differed in the abundance of individual species but that the abundance and richness of species in their communities were similar. *S. tomentosa* invasion led primarily to changes in the quality of Uropodina communities, due to an increase in the shares of species from forest and hygrophilous habitats. Our results suggest that alien plant invasion does not always induce directional changes in mite assemblages, and conclude that the impact of an alien species on Uropodina may cause significant changes in the abundance and richness of individual species without causing significant changes in the abundance and diversity of their community.

**Supplementary Information:**

The online version contains supplementary material available at 10.1007/s10493-024-00951-2.

## Introduction

Invasive plants have strong direct and indirect impacts on invaded ecosystems, particularly due to their effects on the physical and chemical properties of soil substrates (Ehrenfeld 2003; Weidenhamer and Callaway 2010; Gibbons et al. 2017; Stefanowicz et al. 2018). Studies have shown that invasive species increase soil nutrients and increase the rate of edaphic processes such as litter decomposition and mineralization, possibly accelerating nutrient cycling (Vanderhoeven et al. 2005; Liao et al. 2008; Castro-Díez et al. 2014). Some of them affect soil conditions by increasing the activity of soil enzymes (Zhou and Staver 2019). It is also known that alien plants cause changes in soil structure and in nutrient mobilization and/or chelation (Weidenhamer and Callaway 2010; Kalisz et al. 2021), and some secrete allelopathic compounds from the roots (Chengxu et al. 2011; Wardle et al. 2011; Kalisz et al. 2021). Changes in soil properties induced by invasive plants may affect soil biota (Belnap et al. 2005; Zhang et al. 2019). Though it is known that invasive species affect soil microorganisms (Batten et al. 2006; Xiao et al. 2014; McLeod et al. 2016), there is surprisingly little information about the impact of neophytes on soil mesofauna such as mites.

Acari are one of the most abundant microarthropods in the leaf litter and upper soil layers (Rusterholz et al. 2014). This group plays a key role in decomposition and mineralization of organic material (Seastedt 1984), nutrient cycling (Irmler 2000; Wickings and Grandy 2011) and soil formation (Persson 1983). Soil mites, especially taxa that are sensitive to all kinds of soil disturbances, are often used in environmental monitoring (van Straalen 1988; Gulvik 2007), useful in studies on the impact of invasive species on ecosystems.

Most studies report changes in the species composition of Acari in invaded areas (Belnap et al. 2005; Pritekel et al. 2006; McGrath and Binkley 2009; Skubała and Mierny 2009; Christopher and Cameron 2012; Gutiérrez-López et al. 2014; Rusterholz et al. 2014; Kohyt and Skubała 2020), but John et al. (2006), Sterzyńska et al. (2017) and Ustinova et al. (2021) found no influence of invasive plants on mite communities, and that their abundance and species richness in soil under invasive and native species were similar. These various data argue against a clear pattern of the influence of invasive species on mites. Many works report lower numbers of Acari in soil of invaded sites (Belnap et al. 2005; Pritekel et al. 2006; Skubała and Mierny 2009; Motard et al. 2015; Kohyt and Skubała 2020) but other studies show the opposite direction of change: an increase in their number (McGrath and Binkley 2009; Christopher and Cameron 2012; Rusterholz et al. 2014). Hence the impact of invasive plants on Acari populations is hard to predict. Here we studied such effects of invasive steeplebush *Spiraea tomentosa* L. in order to assess the actual interactions of soil fauna with that alien species.

Steeplebush is a North American shrub (Flora of North America 2024; USDA – The Plants Database 2024) which has become one of most successful invasive plants in Europe (Dajdok et al. 2011; Wiatrowska et al. 2020). Its locations were first recorded outside of cultivation in Central Europe at the turn of the 19th/20th centuries (Fiek 1881; Schube 1903). Since that time, *S. tomentosa* has colonized and spread over wetland habitats in eight European countries (Wiatrowska et al. 2020). The shrub has developed many dense monospecific assemblages, especially in wet meadows and transitional and raised bogs (Dajdok et al. 2011; Wiatrowska and Danielewicz 2016), leading to the homogenization of these habitats (Wiatrowska et al. 2023). Steeplebush significantly reduces the number of native plant species (Wiatrowska et al. 2023) and negatively affects several taxonomic groups: spiders (Balkenhol et al. 2018), bees, butterflies and flies, the number and richness of which decrease significantly and linearly with the increase of *S. tomentosa* cover (Wiatrowska et al. 2023). The shrub is characterized by rapid growth (Wiatrowska 2015) and high concentrations of total nonstructural carbohydrates and defence compounds such as condensed tannins and soluble phenols in leaves (Wiatrowska et al. 2018). Its large woody biomass production (Wiatrowska et al. 2022) and its ability to transform plant communities to shrubland may alter the parameters of organic material accumulated as litter on the soil surface and in the upper soil horizon. This in turn may create specific conditions for many saprotrophic taxa associated with organic material, including Uropodina mites, which inhabit leaf litter and the upper soil layers (see e.g. Błoszyk 1983; Karg 1993; Koehler 1997, 1999).

There are 137 mite species of the suborder Uropodina (Acari: Mesostigmata) in Poland (Błoszyk 1999, 2008). Most of them (60%) live in forest soil and litter, while other species (30%) inhabit unstable microhabitats such as dead wood, anthills, bird and mammal nests, and animal feces. Only about 10% occur in open habitats such as meadows, dunes and grasslands (e.g. Błoszyk 1999; Błoszyk et al. 2003; Napierała and Błoszyk 2013). Uropodina mites most often form communities with low species diversity, consisting of a few to a dozen species (Athias-Binche 1981a, b; Błoszyk 1999; Napierała et al. 2009). Uropodina occurs in the greatest abundance in places with a high amount of organic matter such as deciduous forest litter (abundance up to 10,000 specimens/m^2^), dead wood (up to 6,000 specimens/m^2^) and compost (up to 15,000 specimens/m^2^) (Koehler 1997, 1999; Błoszyk et al. 2013, 2016). Many species are saprophagous (Karg 1993) and others are mycetophagous (Faasch 1967; El-Banhawy et al. 1998). Most of the Uropodina known from Poland are species with a narrow range of ecological tolerance (stenobiotic or oligobiotic; 70%); only 6% are characterized by wide tolerance to environmental factors (eurybiotic) (Błoszyk 1999; Błoszyk et al. 2003, 2004). In view of these characteristics, Uropodina mites should be ideal indicators of the effects of steeplebush invasion on soil fauna diversity.

Our literature searches yielded no published information on the impact of *S. tomentosa* on soil biota. In this study we assessed the impact of this invasive shrub on the species number and diversity of mites of the suborder Uropodina in wet meadow soils. We compared the abundance and species richness of Uropodina mites in meadows dominated by *S. tomentosa* and those free of this shrub, and determined whether the structural transformations of wet meadow communities caused by it affected their community composition. Our working hypotheses were that (1) in areas invaded by *S. tomentosa* the abundance and species richness of Uropodina will be lower than in meadows free of this alien species, and that (2) the presence of *S. tomentosa* will transform the structure of Uropodina communities toward more forest-like assemblages. The work was intended to contribute to our understanding of the impact of invasive *S. tomentosa* on the diversity of wet meadow soil fauna.

## Materials and methods

### Study area

The research was carried out in south-western Poland in a wet meadow complex located in the Zgorzelecko-Osiecznicki Forest. The surface formations in the study area are mainly Pleistocene sands and gravels of fluvioglacial or fluvial accumulation, heavily washed by the waters of the melting ice sheet, as well as younger, Holocene patches of fluvial accumulation mud (Rzechowski 1994; Forest Data Bank 2024). The dominant habitats in this area are forest habitats, such as mesic coniferous forest (40%) and mesic mixed coniferous forest (34%) (Góral 2005), between which there are numerous complexes of extensively used meadows and low and transitional bogs.

**Fig. 1:**
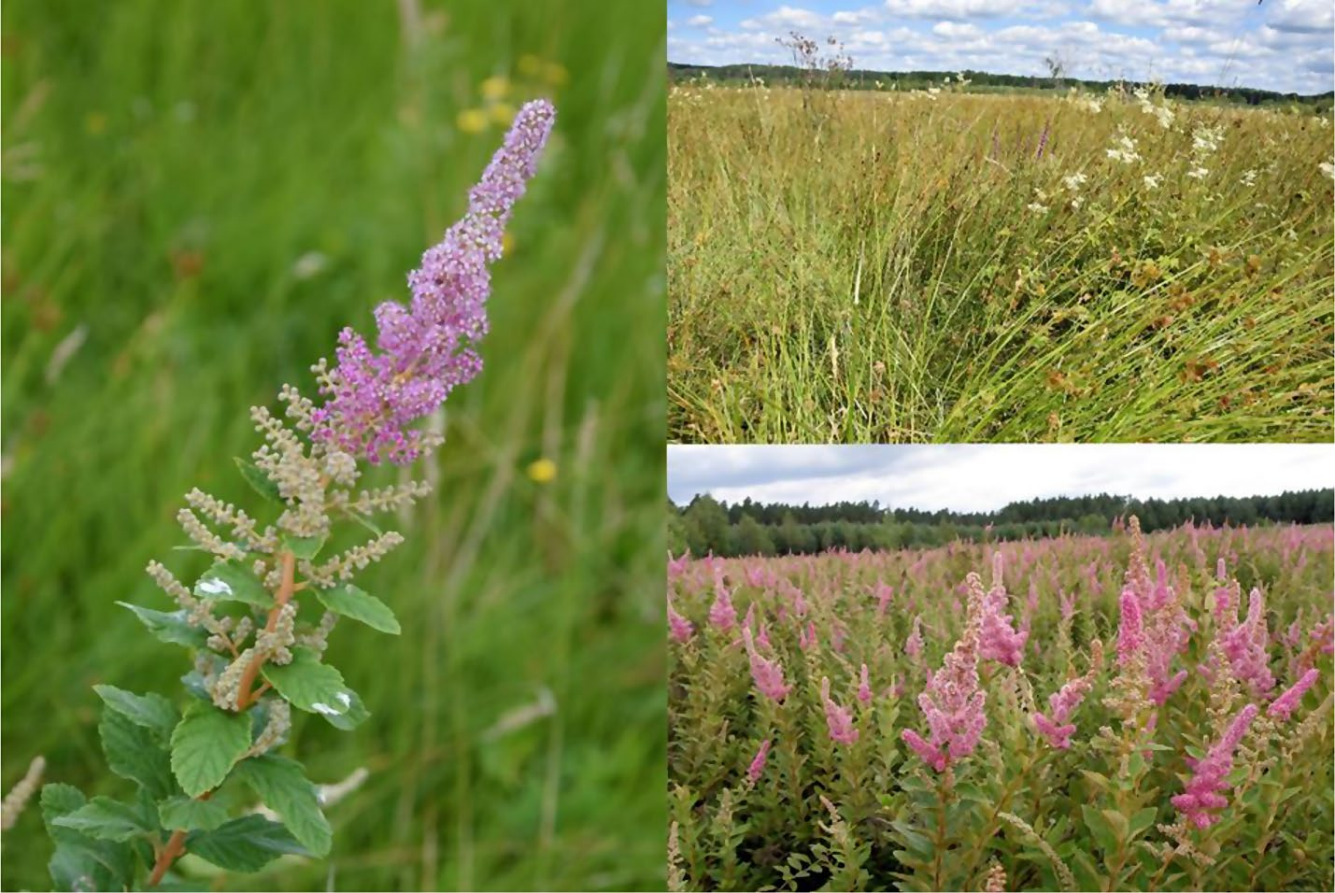
Inflorescence of invasive *Spiraea tomentosa* (**a** – left), and uninvaded (**b** – upper right) and invaded (**c** – lower right) wet meadows in the Lower Silesian Forests (photo by B. Wiatrowska)

Using present vegetation (Forest Data Bank 2024) and hydrological maps (Bieroński et al. 2000), we selected and found forest meadows with soils periodically saturated with water. All the meadows are located in the Lower Silesian Forests mesoregion (Solon et al. 2018) and have a similar geological history (Rzechowski 1994), climate (Woś 1999) and soil properties, the moisture conditions of which are determined by the groundwater table located just below the soil surface (Forest Data Bank 2024). The meadows are dominated by *Juncus effusus* L. and *Molinia caerulea* (L.) Moench, with a significant proportion of grasses: *Deschampsia caespitosa* (L.) P.B., *Holcus lanatus* L., *Calamagrosti canescens* (Weber) Roth, *Agrostis stolonifera* L.; and herbs: *Filipendula ulmaria* (L.) Maxim., *Cirsium palustre* (L.) Scop., *Lythrum salicaria* L. or *Lotus uliginosus* Schkuhr (see Wiatrowska et al. 2023). All these meadows were maintained mainly as part of regular practices, but when mowing was stopped in some of them *S. tomentosa* spread, completely changing the structure and character of the plant communities (Wiatrowska and Danielewicz 2016; Wiatrowska et al. 2023) (Fig. 1).

### Sampling methods

In 2020, eight wet meadow complexes (hereinafter referred to as study sites) were randomly selected for further analyses (Table SI 1). The study sites were at least 1000 ± 830 m apart. Two permanent square plots covering 100 m^2^ were established in each study site: one in a well-preserved meadow with no signs of invasion (“uninvaded plot”), and the other one in a meadow with monodominant stands of *S. tomentosa*, 100% covered by this invasive shrub (“invaded plot”). To exclude or minimize the effect of interaction between invaded and uninvaded plots they were established 50 m apart. Along diagonals of each of the study plots we collected four 0.5 dm^3^ soil samples (sampling core: ø = 8 cm, depth = 10 cm) from organic-mineral horizon A. Soil samples were collected at similar intervals (May, August and December 2020), close to each other but not from the same spot, for a total of 192 soil samples. Eight samples from two plots located in the same study site from the sampling effort in May were excluded because of flood damage. The soil moisture of each sample was determined as the weight ratio between the water content and whole fresh samples (Napierała 2008). The mites were extracted with Tullgren funnels for six days and subsequently preserved in 75% ethanol. Permanent and temporary microscope slide preparations were made (using Hoyer’s medium) and the specimens were identified with the keys of Kadite and Petrova (1977), Evans and Till (1979), Karg (1989), Błoszyk (1999) and Mašán (2001). The samples are deposited in a soil fauna collection in the Natural History Collections of the Faculty of Biology, Adam Mickiewicz University, Poznań.

### Statistical analysis

The abundance and species richness of mites from suborder Uropodina, estimated per soil sample, were tested in relation to three explanatory variables: “plot type” (invaded vs. uninvaded), “sampling period” (May, August, December), and “moisture” as an environmental factor. “Study site” was applied as a random factor. After backward selection of explanatory variables, two were selected for further computations: “plot type” and “sampling period”. “Moisture” as a non-significant factor was excluded from the models, but as it appeared to be a relevant environmental factor for community composition it was applied in ordination analysis. In the second step, to obtain more precise species-specific results the data were analysed separately for the three most abundant uropods (*Olodiscus minima*, *Urodiaspis tecta*, *Trachytes aegrota*) also in relation to “plot type” and “sampling period” as referred to above. Generalized linear mixed-effect (GLMER) models based on a Poisson distribution were applied for analyses. Residuals versus expected values and overdispersion were verified using the DHARMa package (Hartig 2023). In the case of significant deviations in residual diagnostics a negative binomial model was applied. Final results were computed with the ‘Anova’ function using the car package (Fox and Weisberg 2019). Post-hoc analyses were run with the glht function of the multcomp package, applying Tukey’s test (Hothorn et al. 2008). We used the r.squaredGLMM function to calculate marginal *R*^2^_M_ (describes the proportion of variance explained by the fixed factors alone) and conditional *R*^2^_C_ (describes the proportion of variance explained by both the fixed and random factors) (Nakagawa and Schielzeth 2013) of the MuMIn package (Bartoń 2019). Statistical computations were performed with R v4.2.1 (R Core Team 2022).

The analysis of the Uropodina community was based on the indices of dominance (number of individuals of *i*th species compared to individuals of all species in all samples) and frequency (number of samples with *i*th species compared to all samples) (Błoszyk 1999). The dominance and frequency characteristics were computed with data for invaded and uninvaded plots separately, but pooled for all the sampling periods. To test the differences in the structures of dominance, frequency and age between Uropods recorded in invaded and uninvaded plots, the Chi square test (χ^2^) was applied to the analysed data.

Our samples were divided into two datasets depending on plot type (uninvaded, invaded). To assess correlations between communities/clusters presented as different datasets representing different plot types, with moisture as an environmental variable, principal components analysis (PCA, gradient length = 2.7) was performed with CANOCO v.5 (Šmilauer and Lepš 2014). Here the aim of the ordination analysis is to arrange the samples so that those with a similar species composition are located close to each other on the axes, while differing samples are distant from each other.

## Results

### Spatial and seasonal differences in abundance

The analysed material included 482 specimens representing ten species in various developmental stages (adults, protonymphs, deutonymphs) (Table 1). For all species analysed together the only significant effect that determined the number of Uropodina species recorded in soil samples was sampling period (χ^2^ = 7.15, df = 2, *P* = 0.028, Table 2). The lowest number of species was recorded in August (mean ± SE, 1.56 ± 0.38) and did not differ significantly from the number recorded in May (2.36 ± 0.46, Tukey *P* = 0.106) but differed significantly from December (2.06 ± 0.38, Tukey *P* = 0.024). There were no significant differences in the number of Uropodina mite species (χ^2^ = 0.11, df = 1, *P* = 0.742, Table 2) between plot types: invaded, 2.09 ± 0.30; uninvaded, 1.87 ± 0.36. However, the number of individuals was also affected by sampling period (χ^2^ = 15.78, df = 2, *P* < 0.001, Table 2): lowest in August (4.50 ± 1.68) and differing significantly from May (13.86 ± 3.7, Tukey *P* = 0.001) and December (13.50 ± 3.90, Tukey *P* = 0.002). There were no significant differences in the number of individuals (χ^2^ < 0.01, df = 1, *P* = 0.990, Table 2) between plot types: invaded, 8.30 ± 1.72; uninvaded, 12.65 ± 3.43.

**Fig. 2:**
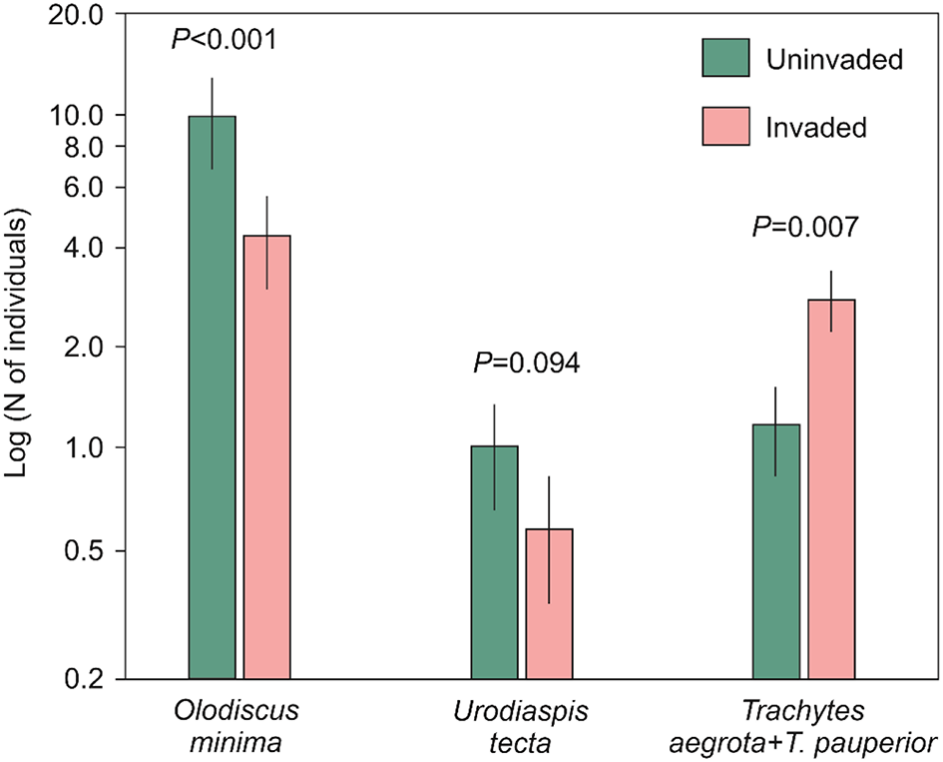
Mean number of individuals (bars), with standard error (whiskers), of the three most abundant taxa of uropods (*Olodiscus minima*, *Urodiaspis tecta*, *Trachytes aegrota* + *T. pauperior*) in relation to uninvaded vs. invaded plots covered by *Spiraea tomentosa*

The most abundant Uropodina mites were *Olodiscus minima*, *Urodiaspis tecta* and *Trachytes* spp. (*Trachytes aegrota* and *T. pauperior* have similar ecology and were pooled together in all analyses, hereafter called *Trachytes* spp.). The number of individuals of those three Uropodina taxa were analysed in relation to plot type and sampling period, showing different patterns in relation to the explanatory variables. The number of *O. minima* and *Trachytes* spp. was significantly dependent on plot type (Fig. 2; Table 2), but only the genus *Trachytes* had higher abundance in invaded plots (2.78 ± 0.59) than in uninvaded plots (1.13 ± 0.34). However, *O. minima* and *U. tecta* were more abundant in open habitats of uninvaded plots (9.83 ± 2.92 and 1.00 ± 0.35 respectively) than in plots invaded by steeplebush (4.26 ± 1.32 and 0.57 ± 0.24 respectively). For sampling period only *O. minima* displayed a significant relation to that variable, reaching minimum abundance in August. The abundance of *U. tecta* was not affected by either of the two fixed effects in the analysis.

**Table 1:** Uropodina species recorded in plots invaded by *Spiraea tomentosa* and in uninvaded meadow plots, with the abundance of adults of both sexes and juvenile stages (D – deutonymphs, P – protonymphs) and habitat preferences (F – forest, O – open habitats). Asterisk indicate source: *Błoszyk (1983)

Species	Invaded				Uninvaded				Habitat preferences*
	Adults		Juveniles		Adults		Juveniles		
	♀♀	♂♂	D	*P*	♀♀	♂♂	D	*P*	
*Olodiscus minima*	67	-	26	5	188	1	31	6	F
*Trachytes aegrota*	33	-	26	5	14	-	11	1	F
*Urodiaspis tecta*	11	-	1	1	14	-	2	7	F
*Uropoda orbicularis*	-	-	1	-	-	-	9	-	F/O
*Trachytes pauperior*	4	-	3	3	3	-	1	-	F
*Iphiduropoda penicillata*	-	-	-	-	-	1	-	-	F
*Oplitis* sp.	-	-	-	-	1	-	-	-	F
*Uroplitella paradoxa*	-	-	-	-	1	-	-	-	O
*Uropoda undulata*	1	2	-	1	-	-	-	-	O/F
*Dinychus perforatus*	1	-	-	-	-	-	-	-	F
No. of individuals	117	2	57	15	221	2	54	14	
No. of species	7			8					

**Table 2:** Relations between dependent variables and fixed effects, showing the response of Uropodina mites to environmental conditions. *P* < 0.05 denoted by asterisk *

Dependent variable					
Number of species (total)					
	Random effect		Variance	SD	
	‘study site’		0.328	0.573	
	Fixed effects	χ^2^	df	*P*	
	‘plot type’	0.11	1	0.742	
	‘period’	7.15	2	0.028*	
			*R*^2^_M_ = 0.036	*R*^2^_C_ = 0.238	
Number of individuals (total)					
	Random effect		Variance	SD	
	‘study site’		0.821	0.906	
	Fixed effects	χ^2^	df	*P*	
	‘plot type’	< 0.01	1	0.990	
	‘period’	15.78	2	< 0.001*	
			*R*^2^_M_ = 0.080	*R*^2^_C_ = 0.278	
Number of individuals of *O. minima*					
	Random effect		Variance	SD	
	‘study site’		3.016	1.737	
	Fixed effects	χ^2^	df	*P*	
	‘plot type’	48.25	1	< 0.001*	
	‘period’	66.28	2	< 0.001*	
			*R*^2^_M_ = 0.166	*R*^2^_C_ = 0.921	
Number of individuals of *U. tecta*					
	Random effect		Variance	SD	
	‘study site’		0.360	0.600	
	Fixed effects	χ^2^	df	*P*	
	‘plot type’	2.81	1	0.094	
	‘period’	2.67	2	0.263	
			*R*^2^_M_ = 0.030	*R*^2^_C_ = 0.094	
Number of individuals of *Trachytes aegrota* + *T. pauperior*					
	Random effect		Variance	SD	
	‘study site’		0.159	0.399	
	Fixed effects	χ^2^	df	*P*	
	‘plot type’	7.39	1	0.007*	
	‘period’	3.64	2	0.162	
			*R*^2^_M_ = 0.065	*R*^2^_C_ = 0.096	

### Dominance and frequency

**Fig. 3:**
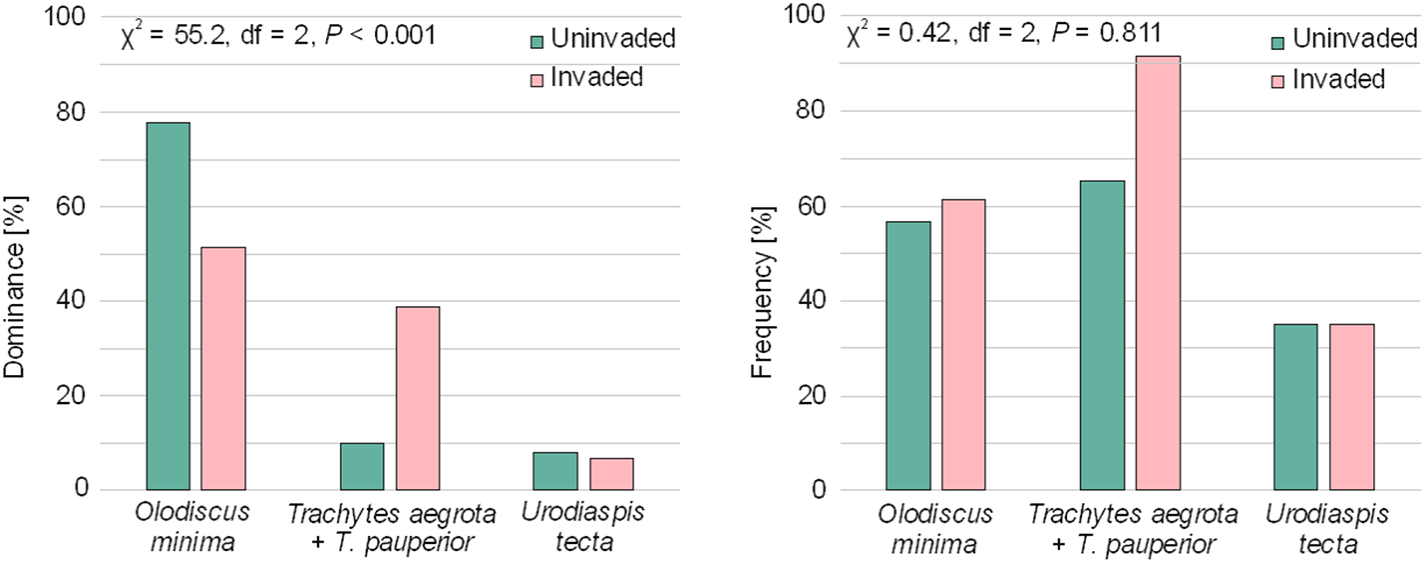
Structures of dominance (left) and frequency (right) of the three most abundant taxa of uropods in relation to plot type – uninvaded vs. invaded by *Spiraea tomentosa*

The Uropodina communities were dominated by the three most numerous taxa in each plot type: *O. minima*, *Trachytes* spp. (*Trachytes aegrota* and *T. pauperior* pooled together) and *U. tecta* (Table 1; Table SI 2), with significant differences in the dominance structure between invaded and uninvaded plots (χ^2^ = 55.2, df = 2, *P* < 0.001, Fig. 3A). In the plots invaded by *S. tomentosa*, *Trachytes* spp. reached higher abundance (38.7%) than in uninvaded plots (10.3%). There was an opposite tendency in the dominance structure for *O. minima*, which was more abundant in uninvaded plots (invaded, 51.3%; uninvaded, 77.7%). The share of *U. tecta* was similar in both plot types (Fig. 3).

The same three taxa were most frequent among the Uropodina communities of both plot types. In invaded plots the share of *Trachytes* spp. was higher (91.3%) than in uninvaded ones (65.2%), and exceeded that of *O. minima* (invaded, 60.9%; uninvaded, 56.5%), but the differences were not significant (χ^2^ = 0.42, df = 2, *P* = 0.811). The share of *U. tecta* was similar in both plot types (Fig. 3).

There were significant differences in the shares of adults and juveniles between plot types. The share of juveniles were significantly higher in invaded (37.7%, *N* = 72) than in uninvaded plots (23.4%, *N* = 68) (χ^2^ = 10.80, df = 1, *P* = 0.001).

### Diversity in community structure

**Fig. 4:**
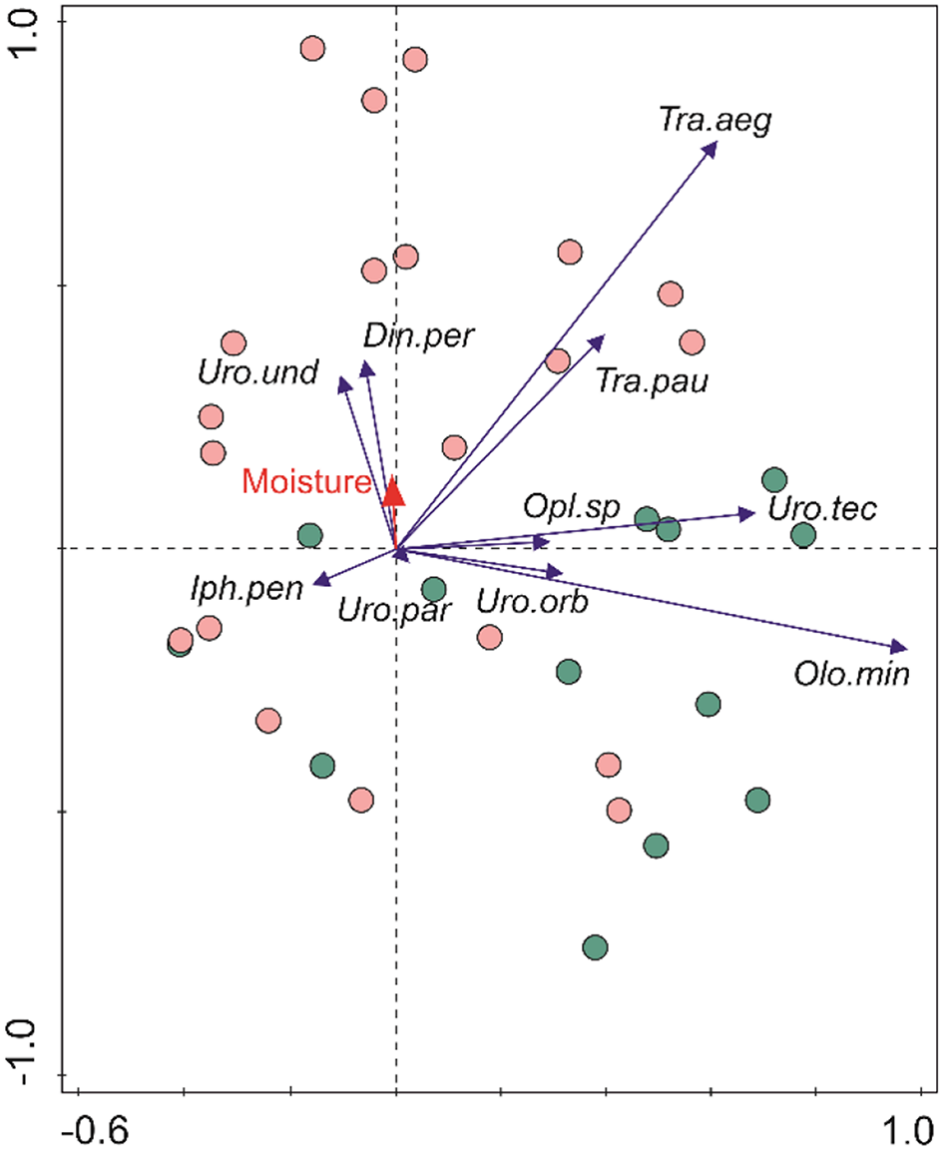
Principal components analysis (PCA) scatterplot showing relationships between Uropodina mite communities from uninvaded and invaded plots on the first two axes. The first and second axes explained 83.7% of variation. Legend: pink dots = plots invaded by *Spiraea tomentosa*; green dots = uninvaded plots. Arrows represent the share of the given species in the assemblage. Species abbreviations: *Din.per = Dinychus perforatus*,* Iph.pen = Iphiduropoda penicillata*,* Olo.min = Olodiscus minima*,* Opl.sp = Oplitis* sp., *Tra.aeg = Trachytes aegrota*,* Tra.pau = T. pauperior*,* Uro.tec = Urodiaspis tecta*,* Uro.par = Uroplitella paradoxa*,* Uro.orb = Uropoda orbicularis*,* Uro.und = U. undulata*

In total, 191 individuals of 7 species were recorded in plots invaded by *S. tomentosa*, in comparison to 291 specimens of 8 species in uninvaded plots. Both groups of samples consisted mostly of the same Uropodina species, common to both uninvaded and invaded plots (e.g., *O. minima*, *T. aegrota*, *T. pauperior*, *U. orbicularis*, *U. tecta*) and forming the core of the whole mite community. PCA analysis showed some qualitative differences between plot types (Fig. 4), manifested in the presence of exclusive species occurring in only one type of plot: *Uropoda undulata* and *Dinychus perforatus* occurred only in plots invaded by *S. tomentosa* (Table 1), whereas *Iphiduropoda penicillata*, *Uroplitella paradoxa* and species of the genus *Oplitis* occurred only in uninvaded plots but their presence was incidental and consisted of only single specimens (Table 1). In the group formed by species common to both plot types the genus *Trachytes* (*T. aegrota* and *T. pauperior*) was more abundant and more frequent in invaded than in uninvaded plots. This group of species formed the upper left part of Fig. 4, representing habitat conditions after the invasion of *S. tomentosa*. *O. minima* and *U. tecta* formed the group preferring uninvaded open habitats (Fig. 4).

## Discussion

The impact of invasive species on microarthropod communities can vary greatly (Belnap et al. 2005), so the direction, strength and effects of these changes must be assessed in each particular context. At least three scenarios are known for the response of mite assemblages after invasion: (i) a decline in the abundance and species richness of mites (Belnap et al. 2005; Pritekel et al. 2006; Skubała and Mierny 2009; Motard et al. 2015; Kohyt and Skubała 2020), (ii) a positive effect on the populations of native microarthropod taxa (McGrath and Binkley 2009; Christopher and Cameron 2012; Rusterholz et al. 2014), or (iii) induction of changes in community composition without significant changes in the number of species or individuals (this study). The latter is relatively rare in the ecology of invasions considered on the soil mesofauna level (Wardle et al. 1995; John et al. 2006; Ustinova et al. 2021).

The microarthropod community in litter is shaped by many factors, such as physical soil properties (L’ubomir et al. 2001; Huhta and Ojala 2006), nutrient availability (King and Hutchinson 1980; Lindbert and Persson 2003), litter complexity (Hansen and Coleman 1998), disturbance (Bird et al. 2004; Reynolds et al. 2007; Rola et al. 2017) or plant diversity (Migge et al. 1998; Hansen 2000). These habitat properties are strongly modified by invasive species (Vanderhoeven et al. 2005; Liao et al. 2008; Castro-Díez et al. 2014). Previous studies have indicated a clear directional impact of alien species on the number and richness of soil mesofauna. Belnap et al. (2005) showed that invasion of *Bromus tectorum* causes a rapid decline in the abundance and richness of microarthropods, whereas Skubała and Mierny (2009) reported a reduction in the abundance and richness of saprophagous and fungivorous mites in areas completely dominated by *Reynoutria sachalinensis* as compared to areas free of this perennial. Lower abundance and richness of mites was found in the litter of alien *Quercus rubra* as compared to litter of the native oak *Q. robur* (Kohyt and Skubała 2020). Increasing density of *Ailanthus altissima* was accompanied by a decrease of litter detritivore abundance (Motard et al. 2015). Other research has shown a negative impact of invasive plant species on the number and abundance of mites. Invasion of *Lonicera maackii* in a deciduous forest had a positive effect on Acari abundance in litter (Christopher and Cameron 2012), and litter under the invasive grass *Microstegium vimineum* in upland forests showed greater abundance of mites than litter from the surrounding forest floor (McGrath and Binkley 2009). According to Rusterholz et al. (2014), in the litter of areas invaded by *Impatiens glandulifera* there were 10–33% more mites than in areas where the plant was removed or in areas not invaded, and this tendency also applied to Uropodina analysed separately. Most studies suggest that the effects of invasion are usually clear and extreme – they illustrate clear negative or positive reactions of indigenous mesofauna communities (Ustinova et al. 2021).

We found that *S. tomentosa* invasion induced some changes in the composition of Uropodina communities, although steeplebush did not have a significant effect on their quantitative characteristics, such as total abundance and species richness. Even though *S. tomentosa* is considered a transforming species (Wiatrowska et al. 2023), the lack of significant changes in the quantitative characteristics of the whole Uropodina mite community means that our first working hypothesis about a clear negative impact of this shrub on the total abundance and species richness of Uropodina should be rejected. Our results are in line with those from other research. Wardle et al. (1995) showed that mite populations generally do not respond to *Senecio jacobaea* infestation in pastures. The abundance of mites was not altered by invasion of *Solidago gigantea* in wet meadows (Sterzyńska et al. 2017) and urban wasteland (Ustinova et al. 2021). Nor were there differences in the number and abundance of mites in areas dominated by native and invasive grass species (John et al. 2006). The effect of an invasive species on mites may be ambiguous and is not always directional. Interpretation of such effects is complicated by the very small number of detailed studies characterizing the ecology and general distribution patterns of mites, including Uropodina. However, some general conclusions can be drawn from research on the impact of invasive species on ecosystems.

It is known that the impact of invasive plants is context-dependent and shaped by the ecological setting (Hejda et al. 2009; Pyšek et al. 2012; Hulme et al. 2013). The structure of soil food webs is controlled by plant inputs and by internal dynamics between trophic levels (Belnap and Phillips 2001), which means that changes in plant cover due to the spread and impact of an alien species can be buffered by habitat properties such as those related to the local structure of the food web in the soil. Wet meadows, like other wetland habitats, are known for their buffering properties (Mander et al. 1996). It is therefore possible that, despite transformations occurring in the meadow ecosystem as a result of *S. tomentosa* invasion, the buffering properties of these habitats (and therefore the amounts of key resources available to mites in the invaded habitats) are similar to those in uninvaded habitats. With an increase in the cover of *S. tomentosa* there is a very significant decrease in plant diversity (Wiatrowska et al. 2023), which, as a consequence of the homogenization of the plant communities, may lead to a reduction in the availability of habitats for Uropodina (Napierała et al. 2006). At the same time, the high biomass production of *S. tomentosa* (Wiatrowska et al. 2022), may increase the food base for mites (or vice versa), and this may help maintain the numbers and species richness of Uropodina.

As a result of steeplebush invasion, two parallel processes can be observed in the Uropodina community: changes in the dominance structure of the most numerous species, and the entry or numerical increase of Uropodina typical of forest habitats (Błoszyk 1983, 1999). A real and rarely analysed effect of the studied invasion seems to be expressed in some qualitative changes in Uropodina mite communities in the habitats invaded by *S. tomentosa*; this allows us to positively verify our second hypothesis, that *S. tomentosa* affects mite community structure. We found that all the studied sites were dominated by Uropodina soil parthenogenetic species, such as *O. minima*,* T. aegrota* and *U. tecta*, which are common and widely distributed in both forest and open areas of Europe (Błoszyk 1983, 1999; Błoszyk et al. 2003). However, the presence of *T. aegrota* and *T. pauperior* was much more marked in invaded plots. Under monodominant stands of *S. tomentosa*,* T. aegrota* reached the eudominant class, and although the differences were not significant, its frequency was much higher there (euconstant). *Trachytes aegrota* is a eurytopic species, found in almost all types of habitats but most frequently and numerously inhabiting forest litter (Błoszyk 1983, 1999). The second species of the genus *Trachytes – T. pauperior –* is also typical of forest habitats (Błoszyk 1983, 1999). The greater abundance of both species and the differences in the dominance structure between invaded and uninvaded sites possibly are promoted by the more “forest-like” features of meadows covered by *S. tomentosa*. It is known that the spread of woody species on grasslands and meadows causes significant changes in the structure of the plant community (Hobbs and Mooney 1986; Costello et al. 2000), resulting, for example, in greater shading of the soil surface (Kudo et al. 2011) and lower daytime temperature (Shen et al. 2022), which leads to changes in arthropod communities (Litt et al. 2014; Woodworth et al. 2020; Lalk et al. 2021). The greater abundance of typical forest dwellers such as *T. pauperior* and *T. aegrota* under *S. tomentosa* indicates slow transformation of the Uropodina communities from those of open-like habitats to more forest-specific ones.

A crucial factor that may affect qualitative differences in the structure of Uropodina communities in invaded and uninvaded sites is moisture content. Although we found that the occurrence of most of the analysed Uropidina species generally was independent of the moisture gradient, two species found only in monodominant stands of *S. tomentosa* showed a preference for wetter habitats than the others. One of them, *U. undulata*, is a rare and sparse hygrophilous species often found in peat bogs, alder forests and riparian forests, while the other, *D. perforatus*, occurs in multi-species deciduous forests and alder forests (Błoszyk 1983, 1999). It seems that, as in the case of other invasive species (McGrath and Binkley 2009), the dense litter layer developed by *S. tomentosa* (Balkenhol et al. 2018) may promote moisture retention, increasing the stability of the habitat (McGrath and Binkley 2009) and favouring hygrophilous species.

Beside moisture changes, the qualitative characteristics of the studied Uropodina communities may also be influenced by significant structural and functional simplification of the plant communities (Wiatrowska et al. 2023) as a result of invasion. In our research we found that such Uropodina mites as *I. penicillata*, a very rare species associated with microhabitats such as rotten stumps, hollows or mammalian nests, and *U. paradoxa*, a species rarely found in xerothermic peat bogs and grasslands (Błoszyk 1983), or *Oplitis* sp., occurred only in meadows free of *S. tomentosa*. It is known that mite communities are strongly determined by the diversity of microhabitats (Hansen 2000; John et al. 2006), and many species of Uropodina, including rare species, are associated with specific microhabitats (Bajerlein and Błoszyk 2004; Napierała et al. 2016, 2021). The homogenization of plant communities caused by the invasion of *S. tomentosa* (Wiatrowska et al. 2023) may limit the number of microhabitats available to rare natives (Seabloom et al. 2006), which often occupy narrow environmental or functional niches (Flather and Sieg 2007).

## Conclusion

The management of invasive plants should always be supported by studies of their impact on the ecosystem (Barney 2016), because their influence is difficult to predict and is not uniform across species (Vilà et al. 2011; Litt et al. 2014; Schirmel et al. 2016). We found that despite profound changes in the ecosystem caused by *S. tomentosa* invasion, the mass spread of the shrub did not lead to significant directional changes in the number and species richness of Uropodina communities living in the leaf litter and upper soil layers of wet meadows. Our results support the suggestion that the structure of aboveground vegetation is not the only factor determining the stability of soil microarthropod communities (John et al. 2006); disturbances caused by the invasion of an alien species may not affect their quantitative characteristics. However, we did find that the invasion of the shrub affected the qualitative features of Uropodina communities. The conditions prevailing in the monodominant stands of *S. tomentosa* seem to favour shade-tolerant and hygrophilous species, initiating a slow transformation process in Uropodina communities toward more forest-like assemblages. Since various environmental factors may be responsible for the observed patterns, these results should be treated with caution. More studies should be done to determine the impact of *S. tomentosa* on microorganisms (bacteria, fungi) and other groups of soil fauna, to yield a more complete picture of the impact of the shrub on soil biota and on the wide range of ecosystem services and the productivity of invaded ecosystems.

## Electronic supplementary material

Below is the link to the electronic supplementary material.

Supplementary Material 1

Supplementary Material 2

## Data Availability

Data is provided within the manuscript, supplementary information or are available from the corresponding author on reasonable request.

## References

[CR1] Athias-Binche F (1981a) Contribution à La connaissance des uropodides libres. Oecologia 193:177–187

[CR2] Athias-Binche F (1981b) Différent types de structures des peuplements d’Uropodides édaphiques de trois écosystèmes forestiers (arachnides: Anactinotriches). Acta Oecol Oecol Gen 2:153–159

[CR3] Bajerlein D, Błoszyk J (2004) Phoresy of Uropoda Orbicularis (Acari: Mesostigmata) by beetles (Coleoptera) associated with cattle dung in Poland. Eur J Entomol 101(1):185–188

[CR4] Balkenhol B, Haase H, Gebauer P, Lehmitz R (2018) Steeplebushes conquer the countryside: influence of invasive plant species on spider communities (Araneae) in former wet meadows. Biodivers Conserv 27:2257e2274. 10.1007/s10531-018-1536-8

[CR5] Barney JN (2016) Invasive plant management must be driven by a holistic understanding of invader impacts. Appl Veg Sci 19(2):183–184. 10.1111/avsc.12239

[CR6] Bartoń K (2019) MuMIn. R package version 1(6)

[CR7] Batten KM, Scow KM, Davies KF, Harrison SP (2006) Two invasive plants alter soil microbial community composition in serpentine grasslands. Biol Invasions 8(2):217–230. 10.1007/s10530-004-3856-8

[CR8] Belnap J, Phillips SL (2001) Soil biota in an ungrazed grassland: response to annual grass (*Bromus tectorum*) invasion. Ecol Appl 11:1261–1275. 10.1890/1051-0761(2001)011[1261:SBIAUG]2.0.CO;2

[CR9] Belnap J, Phillips SL, Sherrod SK, Moldenke A (2005) Soil biota can change after exotic plant invasion: does this affect ecosystem processes? Ecology 86(11):3007–3017. 10.1890/05-0333

[CR10] Bieroński J, Pawlak W, Tomaszewski J (2000) Cartographers. The hydrographic map with commentary. Scale 1:50 000. Sheets: M-33-31-A Pieńsk, M-33-30-B Niesky [map], Pozna&#324

[CR11] Bird SB, Coulson RN, Fisher RF (2004) Changes in soil and litter arthropod abundance following tree harvesting and site preparation in a loblolly Pine (*Pinus taeda* L.) plantation. Ecol Manage 202(1–3):195–208. 10.1016/j.foreco.2004.07.023

[CR12] Błoszyk J (1983) Uropodina of Poland (Acari: Mesostigmata). Dissertationa, Adam Mickiewicz University

[CR13] Błoszyk J (1999) Geographical and ecological diversity of mite communities from the Uropodina cohort (Acari - Mesostigmata) in Poland. In: Uropodina of oak-hornbeam forests (*Carpinion Betuli*). Kontekst, Poznań

[CR14] Błoszyk J (2008) Uropodina. In: Bogdanowicz W, Chudzicka E, Pilipiuk I, Skibińska E (eds) Fauna of Poland - characteristics and checklist of species. Museum and Institute of Zoology at the Polish Academy of Sciences, Warszawa, pp 61–64

[CR16] Błoszyk J, Bajaczyk R, Markowicz M, Gulvik M (2003) Geographical and ecological variability of mites of the suborder Uropodina (Acari: Mesostigmata) in Europe. Biol Lett 40:15–35

[CR15] Błoszyk J, Adamski Z, Napierala A, Dylewska M (2004) Parthenogenesis as a life strategy among mites of the suborder Uropodina (Acari: Mesostigmata). Can J Zool 82:1503–1511. 10.1139/z04-133

[CR18] Błoszyk J, Napierała A, Labijak B, Skwierczyński F, Radtke K, Adamski Z (2013) Dead wood as a winter habitat for Uropodina mites (Acari: Mesostigmata). Badania Fizjograficzne C54:21–28

[CR17] Błoszyk J, Markowicz M, Labijak B, Skwierczyński F, Napierała A (2016) Microgeographic diversity of Uropodina (Acari: Mesostigmata) communities in dead wood and tree hollows. Redia - Giornale Di Zoologia 98:3–12

[CR19] Castro-Díez P, Godoy O, Alonso A, Gallardo A, Saldaña A (2014) What explains variation in the impacts of exotic plant invasions on the nitrogen cycle? A meta-analysis. Ecol Lett 17(1):1–12. 10.1111/ele.12197 24134461 10.1111/ele.12197

[CR20] Chengxu W, Mingxing Z, Xuhui C, Bo Q (2011) Review on allelopathy of exotic invasive plants. Procedia Eng 18:240–246. 10.1016/j.proeng.2011.11.038

[CR21] Christopher CC, Cameron GN (2012) Effects of invasive Amur honeysuckle (*Lonicera maackii*) and white-tailed deer (*Odocoileus virginianus*) on litter-dwelling arthropod communities. Am Midl Nat 167:256–272. 10.1674/0003-0031-167.2.256

[CR22] Costello DA, Lunt ID, Williams JE (2000) Effects of invasion by the indigenous shrub *Acacia sophorae* on plant composition of coastal grasslands in south-eastern Australia. Biol Conserv 6(1):113–121. 10.1016/S0006-3207(00)00058-6

[CR23] Dajdok Z, Nowak A, Danielewicz W, Kujawa-Pawlaczyk J, Bena W (2011) Nobanis – Invasive Alien Species Fact Sheet – *Spiraea tomentosa*. Online Database of the European Network on Invasive Alien Species. https://www.nobanis.org/globalassets/speciesinfo/s/spiraea-tomentosa/spiraea_tomentosa.pdf. Accessed 03 February 2024

[CR24] Ehrenfeld JG (2003) Effects of exotic plant invasions on soil nutrient cycling processes. Ecosystems 6:503–523. 10.1007/s10021-002-0151-3

[CR25] El-Banhawy EM, El-Borolossy MA, El-Sawaf BM, Afia SI (1998) Biological aspects and feeding behaviour of the predacious soil mite *Nenteria Hypotrichus* (Uropodina: Uropodidae). Acarologia 38:357–360

[CR26] Evans GO, Till WM (1979) Mesostigmatic mites of Britain and Ireland (Chelicerata: Acari-Parasitiformes). An introduction to their external morphology and classification. Trans Zool Soc Lond 35:139–270. 10.1111/j.1096-3642.1979.tb00059.x

[CR27] Faasch H (1967) Beitrag Zur Biologie Der Einheimischen Uropodiden Uroobovella marginata, C.L. Koch 1839 und Uropoda Orbicularis, O.F. Müller 1776 und experimentelle Analyse ihres Phoresieverhaltens. Zool Jarhb Syst Bd 94:521–608

[CR28] Fiek E (1881) Flora Von Schlesien preussischen und österreichischen Antheils, enthaltend die wildwachsenden, verwilderten und angebauen Phanerogamen Und Gefasscryptogamen. J. U. Kern’s, Breslau

[CR29] Flather CH, Sieg CH (2007) Species rarity: definition, causes and classification. In: Raphael MG, Molina R (eds) Conservation of rare or little-known species: Biological, social, and economic considerations. Island, Washington, pp 40–66

[CR30] Flora of North America (2024) *Spiraea tomentosa* Linnaeus, http://dev.floranorthamerica.org/Spiraea_tomentosa. Accessed 04 January 2024

[CR31] Forest Data Bank (2024) https://www.bdl.lasy.gov.pl/portal/. Accessed 01 January 2024

[CR32] Fox J, Weisberg S (2019) Nonlinear regression, nonlinear least squares, and nonlinear mixed models in R. Population 150:200

[CR33] Gibbons SM, Lekberg Y, Mummey DL, Sangwan N, Ramsey PW, Gilbert JA (2017) Invasive plants rapidly reshape soil properties in a grassland ecosystem. mSystems 2(2):e00178–e00116. 10.1128/msystems.00178-16 28289729 10.1128/mSystems.00178-16 PMC5340861

[CR34] Góral G (2005) Forests and forest management. In: Fabiszewski J (ed) Nature of Lower Silesia. Polish Academy of Sciences in Wrocław, Wrocław, pp 411–436

[CR35] Gulvik M (2007) Mites (Acari) as indicators of soil biodiversity and land use monitoring: a review. Pol J Ecol 55(3):415–440

[CR36] Gutiérrez-López M, Ranera E, Novo M, Fernández R, Trigo D (2014) Does the invasion of the exotic tree *Ailanthus altissima* affect the soil arthropod community? The case of a riparian forest of the Henares River (Madrid). Eur J Soil Biol 62:39–48. 10.1016/j.ejsobi.2014.02.010

[CR37] Hansen RA (2000) Effects of habitat complexity and composition on a diverse litter microarthropod assemblage. Ecology 81:1120–1132. 10.1890/0012-9658(2000)081[1120:EOHCAC]2.0.CO;2

[CR38] Hansen RA, Coleman DC (1998) Litter complexity and composition are determinants of the diversity and species composition of oribatid mites (Acari: Oribatida) in litterbags. Appl Soil Ecol 9:17–23. 10.1016/S0929-1393(98)00048-1

[CR39] Hartig F (2023) DHARMa: Residual diagnostics for hierarchical (multi-level/mixed) regression models, R package version 0.3.3.0. 10.32614/CRAN.package.DHARMa

[CR40] Hejda M, Pyšek P, Jarošík V (2009) Impact of invasive plants on the species richness, diversity and composition of invaded communities. J Ecol 97(3):393–403. 10.1111/j.1365-2745.2009.01480.x

[CR41] Hobbs RJ, Mooney HA (1986) Community changes following shrub invasion of grassland. Oecologia 70:508–513. 10.1007/BF00379896 28311491 10.1007/BF00379896

[CR42] Hothorn T, Bretz F, Westfall P (2008) Simultaneous inference in General Parametric models. Biom J 50(3):346–363. 10.1002/bimj.200810425 18481363 10.1002/bimj.200810425

[CR43] Huhta V, Ojala R (2006) Collembolan communities in deciduous forests of different origin in Finland. Appl Soil Ecol 31:83–90. 10.1016/j.apsoil.2005.04.001

[CR44] Hulme PE, Pyšek P, Jarošik V, Pergl J, Schaffner U, Vilà M (2013) Bias and error in understanding plant invasion impacts. Trends Ecol Evol 28:12–218. 10.1016/j.tree.2012.10.010 10.1016/j.tree.2012.10.010 23153723

[CR45] Irmler U (2000) Changes in the fauna and its contribution to mass loss and N release during leaf litter decomposition in two deciduous forests. Pedobiologia 44:105–118. 10.1078/S0031-4056(04)70032-3

[CR46] John MGS, Wall DH, Hunt HW (2006) Are soil mite assemblages structured by the identity of native and invasive alien grasses? Ecology 87(5):1314–24. http://doi.10.1890/0012-9658(2006)87 10.1890/0012-9658(2006)87[1314:asmasb]2.0.co;2 16761609

[CR47] Kadite BA, Petrova AD (1977) Kogorta Trachytina, sem. Trachytidae. Izdatelstvo Nauka, Leningrad

[CR48] Kalisz S, Kivlin SN, Bialic-Murphy L (2021) Allelopathy is pervasive in invasive plants. Biol Inv 23(2):367–371. 10.1007/s10530-020-02383-6

[CR49] Karg W (1989) Acari (Acarina), Milben, Unterordnung Parasitiformes (Anactinochaeta) Uropodina Kramer, Schildkrötenmilben. Gustav Fischer, Jena

[CR50] Karg W (1993) Acari (Acarina), Milben Parasitiformes (Anactinochaeta) Cohors Gmasina Leach, Raubmilben. Die Tierwelt Deutschlands. Gustav Fischer, Jena

[CR51] King KL, Hutchinson KJ (1980) Effects of superphosphate and stocking intensity on grassland microarthropods. J Appl Ecol 17(3):581–591. 10.2307/2402638

[CR52] Koehler HH (1997) Mesostigmata (Gamasina, Uropodina), efficient predators in agroecosystems. Agric Ecosyst Environ 62:105–117. 10.1016/S0167-8809(96)01141-3

[CR53] Koehler HH (1999) Predatory mites (Gamasina, Mesostigmata). Agric Ecosyst Environ 74:395–410. 10.1016/S0167-8809(99)00045-6

[CR54] Kohyt J, Skubała P (2020) Oribatid mite (Acari: Oribatida) communities reveal the negative impact of the red oak (*Quercus rubra* L.) on soil fauna in Polish commercial forests. Pedobiologia 79:150594. 10.1016/j.pedobi.2019.150594

[CR55] Kudo G, Amagai Y, Hoshino B, Kaneko M (2011) Invasion of dwarf bamboo into alpine snow-meadows in northern Japan: pattern of expansion and impact on species diversity. Ecol Evol 1(1):85–96. 10.1002/ece3.9 22393485 10.1002/ece3.9 PMC3287379

[CR56] L’ubomir K, Luptacik P, Miklisova D, Mati R (2001) Soil oribatida and collembola communities across a land depression in an arable field. Eur J Soil Biol 37(4):285–289. 10.1016/S1164-5563(01)01106-2

[CR57] Lalk S, Hartshorn J, Coyle DR (2021) Invasive woody plants and their effects on arthropods in the United States: challenges and opportunities. Ann Entomol Soc Am 114(2):192–205. 10.1093/aesa/saaa054

[CR58] Liao C, Peng R, Luo Y, Zhou X, Wu X, Fang C, Chen J, Li B (2008) Altered ecosystem carbon and nitrogen cycles by plant invasion: a meta-analysis. New Phytol 177(3):706–714. 10.1111/j.1469-8137.2007.02290.x 18042198 10.1111/j.1469-8137.2007.02290.x

[CR59] Lindbert N, Persson T (2003) Effects of long-term nutrient fertilization and irrigation on the microarthropod community in a boreal Norway Spruce stand. Ecol Manage 188(1–3):125–135. 10.1016/j.foreco.2003.07.012

[CR60] Litt AR, Cord EE, Fulbright TE, Schuster GL (2014) Effects of invasive plants on arthropods. Conserv Biol 28(6):1532–1549. http://doi.10.1111/cobi.12350 10.1111/cobi.12350 25065640

[CR61] Mander Ü, Lõhmus K, Kuusemets V, Ivask M (1996) The potential role of wet meadows and grey alder forests as buffer zones. In Int. Conf. Buffer Zones: Their Processes and Potential in Water Protection, Woodstock, Oxfordshire(UK), 30 Aug-2 Sep 1996 (Vol. 1996, pp. 24–25)

[CR62] Mašán P (2001) Roztoce Kohorty Uropodina (Acari, Mesostigmata) Slovenska. Annot Zool Bot 223:1–320

[CR63] McGrath DA, Binkley MA (2009) *Microstegium vimineum* invasion changes soil chemistry and microarthropod communities in Cumberland Plateau forests. Southeast Nat 8:141–156. 10.1656/058.008.0113

[CR64] McLeod ML, Cleveland CC, Lekberg Y, Maron JL, Philippot L, Bru D, Callaway RM (2016) Exotic invasive plants increase productivity, abundance of ammonia-oxidizing bacteria and nitrogen availability in intermountain grasslands. J Ecol 104(4):994–1002. 10.1111/1365-2745.12584

[CR65] Migge S, Maraun M, Scheu S, Schaefer M (1998) The oribatid mite community (Acarina) of pure and mixed stands of beech (*Fagus sylvatica*) and spruce (*Picea abies*) of different age. Appl Soil Ecol 9(1–3):115–121. 10.1016/S0929-1393(98)00065-1

[CR66] Motard E, Dusz S, Geslin B, Akpa-Vinceslas M, Hignard C, Babiar O, Clair-Maczulajtys D, Michel-Salzat A (2015) How invasion by *Ailanthus altissima* transforms soil and litter communities in a temperate forest ecosystem. Biol Invasions 17:1817–1832. 10.1007/s10530-014-0838-3

[CR67] Nakagawa S, Schielzeth H (2013) A general and simple method for obtaining R2 from generalized linear mixed-effects models. Methods Ecol Evol 4(2):133–142

[CR68] Napierała A (2008) Group structure and spatial distribution of Uropodina (Acari: Mesostigmata) in selected forest complexes of Wielopolska. PhD Thesis, Adam Mickiewicz University, Poznań, Poland

[CR69] Napierała A, Błoszyk J (2013) Unstable microhabitats (merocenoses) as specific habitats of Uropodina mites (Acari: Mesostigmata). Exp Appl Acarol 60(2):163–80. http://doi.10.1007/s10493-013-9659-9 10.1007/s10493-013-9659-9 PMC3641307 23539262

[CR71] Napierała A, Błoszyk J, Kozak J, Bruin J (2006) Spatial distribution of mites of the suborder Uropodina (Acari: Mesostigmata) in a small isolated forest area. Exp Appl Acarol 39(3):289–295. 10.1007/s10493-006-9007-4 16804769 10.1007/s10493-006-9007-4

[CR70] Napierała A, Błoszyk J, Bruin J (2009) Communities of uropodine mites (Acari: Mesostigmata) in selected oak-hornbeam forests of the Wielkopolska region (Poland). Exp Appl Acarol 49(4):291–303. http://doi.10.1007/s10493-009-9262-2 10.1007/s10493-009-9262-2 19326248

[CR72] Napierała A, Mądra A, Leszczyńska-Deja K, Gwiazdowicz DJ, Gołdyn B, Błoszyk J (2016) Community structure variability of Uropodina mites (Acari: Mesostigmata) in nests of the common mole, Talpa europaea, in Central Europe. Exp Appl Acarol 68(4):429–440. 10.1007/s10493-016-0017-6 26861069 10.1007/s10493-016-0017-6 PMC4783448

[CR73] Napierała A, Maziarz M, Hebda G, Broughton RK, Rutkowski T, Zacharyasiewicz M, Błoszyk J (2021) Lack of specialist nidicoles as a characteristic of mite assemblages inhabiting nests of the ground-nesting wood warbler, *Phylloscopus sibilatrix* (Aves: Passeriformes). Exp Appl Acarol 84(1):149–170. http://doi.10.1007/s10493-021-00620-8 10.1007/s10493-021-00620-8 PMC8102295 33939099

[CR74] Persson T (1983) Influence of soil animals on nitrogen mineralization in a northern scots pine forest. In: Lebrun P, André H, De-Medts A, Grégoire-Wibo C, Wauthy G (eds) Belgium New Trend in Soil Biology. Dieu Brichart, Louvain-La-Neuve, pp 117–126

[CR75] Pritekel C, Whittemore-Olson A, Snow N, Moore JC (2006) Impacts from invasive plant species and their control on the plant community and belowground ecosystem at Rocky Mountain National Park, USA. Appl Soil Ecol 32(1):132–141. 10.1016/j.apsoil.2005.01.010

[CR76] Pyšek P, Jarošík V, Hulme PE, Pergl J, Hejda M, Schaffner U, Vilá M (2012) A global assessment of invasive plant impacts on resident species, communities and ecosystems: the interaction of impact measures, invading species’ traits and environment. Glob Chang Biol 18:1725–1737. 10.1111/j.1365-2486.2011.02636.x

[CR77] R Core Team (2022) R: A language and environment for statistical computing. R Foundation for Statistical Computing, Vienna, Austria. URL https://www.R-project.org/

[CR78] Reynolds BC, Hamel J, Isbanloly J, Klausman L, Moorhead KK (2007) From forest to Fen: Microarthropod abundance and litter decomposition in a southern Appalachian floodplain/fen complex. Pedobiologia 51(4):273–280. 10.1016/j.pedobi.2007.04.006

[CR79] Rola K, Kurek P, Skubała P (2017) Badger (*Meles meles*) disturbances affect oribatid mite (Acari: Oribatida) communities in European temperate forests. Appl Soil Ecol 121:20–30. 10.1016/j.apsoil.2017.09.013

[CR80] Rusterholz HP, Salamon JA, Ruckli R, Baur B (2014) Effects of the annual invasive plant *Impatiens glandulifera* on the Collembola and Acari communities in a deciduous forest. Pedobiologia 57(4–6):285–291. 10.1016/j.pedobi.2014.07.001

[CR81] Rzechowski J (1994) Cartographers. Surface geological formations. Scale 1: 1,500,000. Worksheet 21.1. [map]. E. Romera Polish Cartographic Publishers S.A, Warsaw

[CR82] Schirmel J, Bundschuh M, Entling MH, Kowarik I, Buchholz S (2016) Impacts of invasive plants on resident animals across ecosystems, taxa, and feeding types: a global assessment. Glob Chang Biol 22(2):594–603. http://doi.10.1111/gcb.13093 10.1111/gcb.13093 26390918

[CR83] Schube T (1903) Die Verbreitung Der Gefasspflanzen in Schlesien preussischen und osterreichischen antheils. Druck Von. R. Nischkovsky, Breslau

[CR84] Seabloom EW, Williams JW, Slayback D, Stoms DM, Viers JH, Dobson AP (2006) Human impacts, plant invasion, and imperiled plant species in California. Ecol Appl 16(4):1338–50. http://doi.10.1890/1051-0761(2006)016[1338:hipiai]2.0.co;2 10.1890/1051-0761(2006)016[1338:hipiai]2.0.co;2 16937802

[CR85] Seastedt TR (1984) The role of microarthropods in decomposition and mineralization processes. Annu Rev Entomol 29(1):25–46. 10.1146/annurev.en.29.010184.000325

[CR86] Shen X, Liu Y, Liu B, Zhang J, Wang L, Lu X, Ijang M (2022) Effect of shrub encroachment on land surface temperature in semi-arid areas of temperate regions of the Northern Hemisphere. Agric Meteorol 320:108943. 10.1016/j.agrformet.2022.108943

[CR87] Skubała P, Mierny A (2009) Invasive Reynoutria taxa as a contaminant of soil. Does it reduce abundance and diversity of microarthropods and damage soil habitat? Pesticides 1–4:57–62

[CR88] Šmilauer P, Lepš J (2014) Multivariate analysis of ecological data using CANOCO 5. Cambridge University Press, Cambridge

[CR89] Solon J, Borzyszkowski J, Bidłasik M, Richling A, Badora K, Balon J, Brzezińska-Wójcik T, Chab Ł, Dobrowolski R, Grzegorczyk I, Jodłowski M, Kistowski M, Kot R, Krąż P, Lechnio J, Macias A, Majchrowska A, Malinowska E, Migoń P, Ziaja W (2018) Physico-geographical mesoregions of Poland: Verification and adjustment of boundaries on the basis of contemporary spatial data. Geogr Pol 91(2):143–170. 10.7163/GPol.0115

[CR90] Stefanowicz AM, Majewska ML, Stanek M, Nobis M, Zubek S (2018) Differential influence of four invasive plant species on soil physicochemical properties in a pot experiment. J Soils Sediments 18(4):1409–1423. 10.1007/s11368-017-1873-3

[CR91] Sterzyńska M, Shrubovych J, Nicia P (2017) Impact of plant invasion (*Solidago gigantea* L.) on soil mesofauna in a riparian wet meadows. Pedobiologia 64:1–7. 10.1016/j.pedobi.2017.07.004

[CR92] USDA – The Plants Database (2024) Fact Sheet – *Spiraea tomentosa*. https://plants.usda.gov/home/classification/91846. Accessed 20 January 2024

[CR93] Ustinova EN, Schepetov DM, Lysenkov SN, Tiunov AV (2021) Soil arthropod communities are not affected by invasive *Solidago gigantea* Aiton (Asteraceae), based on morphology and metabarcoding analyses. Soil Biol Biochem 159:108288. 10.1016/j.soilbio.2021.108288

[CR94] van Straalen NM (1988) Evaluation of bioindicator systems derived from soil arthropod communities. Appl Soil Ecol 9(1–3):429–437. 10.1016/S0929-1393(98)00101-2

[CR95] Vanderhoeven S, Dassonville N, Meerts P (2005) Increased topsoil mineral nutrient concentrations under exotic invasive plants in Belgium. Plant Soil 275(1):169–179. 10.1007/s11104-005-1257-0

[CR96] Vilà M, Espinar JL, Hejda M, Hulme PE, Jarošík V, Maron JL, Pergl J, Schaffner U, Sun Y, Pyšek P (2011) Ecological impacts of invasive alien plants: a meta-analysis of their effects on species, communities and ecosystems. Ecol Lett 14(7):702–8. http://doi.10.1111/j.1461-0248.2011.01628.x 10.1111/j.1461-0248.2011.01628.x 21592274

[CR98] Wardle DA, Nicholson KS, Rahman A (1995) Ecological effects of the invasive weed species *Senecio jacobaea* L. (Ragwort) in a New Zealand pasture. Agri Eco Enviro 56(1):19–28. 10.1016/0167-8809(95)00636-2

[CR97] Wardle DA, Karban R, Callaway RM (2011) The ecosystem and evolutionary contexts of allelopathy. Trends Ecol Evol 26(12):655–662. 10.1016/j.tree.2011.08.003 21920626 10.1016/j.tree.2011.08.003

[CR99] Weidenhamer JD, Callaway RM (2010) Direct and indirect effects of invasive plants on soil chemistry and ecosystem function. J Chem Ecol 36(1):59–69. http://doi.10.1007/s10886-009-9735-0 10.1007/s10886-009-9735-0 20077127

[CR100] Wiatrowska B (2015) Determinants of the invasion of the steeplebush (*Spiraea tomentosa* L.) in the Lower Silesian Forests. Dissertation. Poznan University of Life Sciences

[CR101] Wiatrowska B, Danielewicz W (2016) Environmental determinants of the steeplebush (*Spiraea tomentosa* L.) invasion in the Bory Dolnosląskie Forest. Sylwan 160(8):696–704. http://sylwan.ibles.waw.pl/

[CR103] Wiatrowska B, Łukowski A, Karolewski P, Danielewicz W (2018) Invasive *Spiraea tomentosa*: a new host for monophagous *Earias clorana*? Arthropod Plant Interact 12(3):423–434. 10.1007/s11829-017-9592-7

[CR104] Wiatrowska B, Pietras M, Kolanowska M, Danielewicz W (2020) Current occurrence and potential future climatic niche distribution of the invasive shrub *Spiraea tomentosa* L. in its native and non-native ranges. Glob Ecol Conserv 24:01226. 10.1016/j.gecco.2020.e01226

[CR105] Wiatrowska B, Wawro A, Gieparda W, Waliszewska B (2022) Bioethanol Production Potential and Other Biomass Energy Properties of Invasive *Reynoutria, Solidago*, and *Spiraea* plants. Forests 3(10):1582. 10.1016/j.gecco.2020.e01226

[CR102] Wiatrowska B, Kurek P, Moroń D, Celary W, Chrzanowski A, Trzciński P, Piechnik Ł (2023) Linear scaling negative effects of invasive Spiraea tomentosa (Rosaceae) on wetland plants and pollinator communities. NeoBiota 81:63–90. 10.3897/neobiota.81.95849

[CR106] Wickings K, Grandy AS (2011) The oribatid mite *Scheloribates moestus* (Acari: Oribatida) alters litter chemistry and nutrient cycling during decomposition. Soil Biol Biochem 43(2):351–358. 10.1016/j.soilbio.2010.10.023

[CR107] Woodworth GR, Ward JN, Carr DE (2020) Exotic tree and shrub invasions alter leaf-litter microflora and arthropod communities. Oecologia 93(1):177–187. http://doi.10.1007/s00442-020-04657-1 10.1007/s00442-020-04657-1 32322986

[CR108] Woś A (1999) Climate of Poland. Polish Scientific Publishers PWN, Warsaw

[CR109] Xiao HF, Feng YL, Schaefer DA, Yang XD (2014) Soil fungi rather than bacteria were modified by invasive plants, and that benefited invasive plant growth. Plant Soil 378(1):253–264. 10.1007/s11104-014-2040-x

[CR110] Zhang P, Li B, Wu J, Hu S (2019) Invasive plants differentially affect soil biota through litter and rhizosphere pathways: a meta-analysis. Ecol Lett 22(1):200–210. http://doi.10.1111/ele.13181 10.1111/ele.13181 30460738

[CR111] Zhou Y, Staver AC (2019) Enhanced activity of soil nutrient-releasing enzymes after plant invasion: a meta-analysis. Ecology 100(11):e02830. http://doi.10.1002/ecy.2830 10.1002/ecy.2830 31323119

