# Supplementary material for: Consequences of Spiraea tomentosa invasion in Uropodina mite (Acari: Mesostigmata) communities in wet meadows
Source: Exp Appl Acarol. 2024 Aug 1;93(3):609–26. doi: 10.1007/s10493-024-00951-2 (PMC11464589; doi:10.1007/s10493-024-00951-2)
Supplement: Supplementary file 1 — Supplementary Material 1 [file 10493_2024_951_MOESM1_ESM.pdf]

Consequences of *Spiraea tomentosa* invasion on changes in Uropodina (Acari: Mesostigmata) mite communities from wet meadows

Experimental and Applied Acarology

Blanka Wiatrowska[1*], Przemysław Kurek[2], Tomasz Rutkowski[3,4**], Agnieszka Napierała[4***], Paweł Sienkiewicz[5], Jerzy Błoszyk[3,4****]

[1]Department of Botany and Forest Habitats, Poznań University of Life Sciences, Wojska Polskiego 71 D, 60-625, Poznan, Poland. ORCID: 0000-0003-2542-4953

[2]Department of Plant Ecology and Environmental Protection, Adam Mickiewicz University, Uniwersytetu Poznańskiego 6, 61-614 Poznań, Poland. ORCID: 0000-0002-5366-3057

[3]Natural History Collections, Adam Mickiewicz University, Uniwersytetu Poznańskiego 6, 61-614, Poznań, Poland. [**]ORCID: 0000-0002-1565-7473 [****]ORCID: 0000-0002-3615-226

[4]Department of General Zoology, Adam Mickiewicz University, Uniwersytetu Poznańskiego 6, 61-614, Poznań, Poland. [***]ORCID: 0000-0002-9540-4600

[5]Department of Entomology and Environmental Protection, Poznań University of Life Sciences, ul. Dąbrowskiego 159, 60-594, Poznań, Poland. ORCID: 0000-0003-4714-8873

[*]Corresponding author: blanka.wiatrowska@up.poznan.pl

Supplementary Information (SI 1)

Table SI 1 List of study sites.

Study sites	The nearest village	Plot type	Geographical coordinates (DD)	
1	Stary Węgliniec	invaded	51.299308	15.185377
	Stary Węgliniec	uninvaded	51.298531	15.185658
2	Ruszów	invaded	51.394345	15.157955
	Ruszów	uninvaded	51.394706	15.157473
3	Ruszów	invaded	51.391697	15.158341
	Ruszów	uninvaded	51.391937	15.158308
4	Ruszów	invaded	51.391576	15.162922
	Ruszów	uninvaded	51.391857	15.162810
5	Polana	invaded	51.396868	15.137064
	Polana	uninvaded	51.396800	15.136821
6	Parowa	invaded	51.380197	15.228287
	Parowa	uninvaded	51.380118	15.228767
7	Parowa	invaded	51.378467	15.232805
	Parowa	uninvaded	51.378830	15.232717
8	Parowa	invaded	51.378973	15.230439
	Parowa	uninvaded	51.379290	15.231869
