# Supplementary material for: Consequences of Spiraea tomentosa invasion in Uropodina mite (Acari: Mesostigmata) communities in wet meadows
Source: Exp Appl Acarol. 2024 Aug 1;93(3):609–26. doi: 10.1007/s10493-024-00951-2 (PMC11464589; doi:10.1007/s10493-024-00951-2)
Supplement: Supplementary file 2 — Supplementary Material 2 [file 10493_2024_951_MOESM2_ESM.pdf]

Consequences of *Spiraea tomentosa* invasion in Uropodina mite (Acari: Mesostigmata) communities in wet meadows

Experimental and Applied Acarology

Blanka Wiatrowska[1*], Przemysław Kurek[2], Tomasz Rutkowski[3,4**], Agnieszka Napierała[4***], Paweł Sienkiewicz[5], Jerzy Błoszyk[3,4****]

[1]Department of Botany and Forest Habitats, Poznań University of Life Sciences, Wojska Polskiego 71D, 60-625, Poznan, Poland. ORCID: 0000-0003-2542-4953

[2]Department of Plant Ecology and Environmental Protection, Adam Mickiewicz University, Uniwersytetu Poznańskiego 6, 61-614 Poznań, Poland. ORCID: 0000-0002-5366-3057

[3]Natural History Collections, Adam Mickiewicz University, Uniwersytetu Poznańskiego 6, 61-614, Poznań, Poland. [**]ORCID: 0000-0002-1565-7473 [****]ORCID: 0000-0002-3615-226

[4]Department of General Zoology, Adam Mickiewicz University, Uniwersytetu Poznańskiego 6, 61-614, Poznań, Poland. [***]ORCID: 0000-0002-9540-4600

[5]Department of Entomology and Environmental Protection, Poznań University of Life Sciences, ul. Dąbrowskiego 159, 60-594, Poznań, Poland. ORCID: 0000-0003-4714-8873

[*]Corresponding author: blanka.wiatrowska@up.poznan.pl

Supplementary Information (SI 2)

Table SI 2 Uropodina species recorded in invaded and uninvaded plots with number of individuals (N), their dominance D [%] and frequency F [%].

Species	Invaded			Uninvaded		
	N	D [%]	F [%]	N	D [%]	F [%]
Olodiscus minima (Kramer, 1882)	98	51.3	60.9	226	77.7	56.5
Trachytes aegrota (C.L. Koch, 1841)	64	33.5	69.6	26	9.0	52.2
Urodiaspis tecta (Kramer, 1876)	13	6.8	34.8	23	7.9	34.8
Uropoda orbicularis (Müller, 1776)	1	0.5	4.3	9	3.1	17.4
Trachytes pauperior (Berlese, 1914)	10	5.3	21.7	4	1.4	13.0
Iphiduropoda penicillata (Hirschmann et Z.-	0	0	0	1	0.3	4.3

Nicol, 1961)						
Oplitis sp.	0	0	0	1	0.3	4.3
Uroplitella paradoxa (Canestrini et Berlese, 1884)	0	0	0	1	0.3	4.3
Uropoda undulata (Hirschmann et Zirngiebl-Nicol, 1969)	4	2.1	13.0	0	-	-
Dinychus perforatus (Kramer, 1882)	1	0.5	4.3	0	-	-
Total	191			291		
